# Impact of osteopenia and osteosarcopenia on the outcomes after surgery of hepatobiliary-pancreatic cancers

**DOI:** 10.3389/fonc.2024.1403822

**Published:** 2024-07-19

**Authors:** Xiaofeng Wang, Min Wu, Qian Liu, Wei He, Yong Tian, Yan Zhang, Cuiping Li, Yanni Liu, Anqi Yu, Hongyan Jin

**Affiliations:** ^1^ Department of Oncology, Wuhan Puren Hospital Affiliated to Wuhan University of Science and Technology, Wuhan, Hubei, China; ^2^ Department of Oncology, Third People’s Hospital of Honghe Prefecture, Gejiu, China

**Keywords:** osteopenia, osteosarcopenia, pancreatic cancer, biliary tract neoplasms, hepatocellular carcinoma (HCC)

## Abstract

**Objective:**

The purpose of this study is to investigate potential associations between osteopenia, osteosarcopenia, and postoperative outcomes in patients with hepatobiliary-pancreatic cancer (HBPC).

**Methods:**

Three online databases, including Embase, PubMed, and the Cochrane Library, were thoroughly searched for literature describing the relationship between osteopenia, osteosarcopenia, and outcomes of surgical treatment of HBPC patients from the start of each database to September 29, 2023. The Newcastle-Ottawa Scale was used to rate the quality of the studies.

**Results:**

This analysis included a total of 16 articles with a combined patient cohort of 2,599 individuals. The results demonstrated that HBPC patients with osteopenia had significantly inferior OS (HR: 2.27, 95% CI: 1.70-3.03, *p* < 0.001) and RFS (HR: 1.96, 95% CI: 1.42-2.71, *p* < 0.001) compared to those without osteopenia. Subgroup analysis demonstrated that these findings were consistent across univariate and multivariate analyses, as well as hepatocellular carcinoma, biliary tract cancer, and pancreatic cancer. The risk of postoperative major complications was significantly higher in patients with osteopenia compared to those without osteopenia (OR: 1.66, 95% CI: 1.19-2.33, *p* < 0.001). Besides, we also found that the presence of osteosarcopenia in HBPC patients was significantly related to poorer OS (HR: 3.31, 95% CI: 2.00-5.48, *p* < 0.001) and PFS (HR: 2.50, 95% CI: 1.62-3.84, *p* < 0.001) in comparison to those without osteosarcopenia.

**Conclusion:**

Preoperative osteopenia and osteosarcopenia can predict poorer OS and RFS with HBPC after surgery.

## 1 Introduction

Hepatobiliary-pancreatic cancer (HBPC) includes pancreatic cancer (PC), gallbladder cancer, biliary tract cancer (BTC), and liver cancer (1–3). In 2018, an estimated 1.85 million novel instances of HBPC were diagnosed on a global scale. This accounted for 10% of the total count of freshly documented malignancies, thereby exerting substantial economic pressures (4, 5). The absence of early symptoms and reliable diagnostic markers often leads patients to present at an advanced stage, severely limiting available treatment options (6, 7). Although surgical intervention persists as the solitary potential curative recourse for individuals afflicted by HBPC, a substantial proportion of patients receive a belated diagnosis, rendering them ineligible for surgical interventions. Consequently, the 5-year survival rate for those with resectable tumors remains conspicuously modest (6, 7). The persistence of tumor recurrence and a diminished postoperative survival rate continues to pose substantial impediments in the management of HBPC patients. In order to refine the preoperative identification of suitable surgical candidates and optimize the trade-off between surgical risks and anticipated survival gains, it becomes imperative to discern innovative risk factors associated with unfavorable clinical outcomes.

Though not as profound as in osteoporosis, osteopenia is marked by a reduced bone mineral density (BMD) in comparison to the standard (8). Dual-energy X-ray absorptiometry (DXA) remains the conventional method for BMD assessment; however, a prevailing shift is observed towards employing computed tomography (CT) scan-derived attenuation values to ascertain BMD levels. This is particularly prevalent in oncological patients, as CT scans are commonly used for preoperative evaluation or postoperative recurrence monitoring in such patients (9). About 80% of elderly cancer patients had osteopenia or osteoporosis, according to a recent study (10). Furthermore, it’s common for sarcopenia and osteopenia to co-occur in older adults (11). Osteosarcopenia, characterized by the concurrent presence of osteopenia and sarcopenia, represents a relatively recent conceptualization (12). Several systematic reviews have elucidated the impact of sarcopenia, encompassing diminished muscle quantity and quality, on postoperative outcomes following hepatobiliary-pancreatic surgeries (13–15). However, the impact of preoperative osteopenia or osteosarcopenia on postoperative outcomes for hepatobiliary-pancreatic cancer surgery remains controversial.

Consequently, the primary objective of this study was to evaluate the prognostic significance of preoperative osteopenia or osteosarcopenia in HBPC patients subjected to surgical resection.

## 2 Methods

### 2.1 Search strategy

Commencing on September 29, 2023, a computerized search of various bibliographic databases, including EMBASE, Cochrane Library, and PubMed, was initiated. This comprehensive search utilized specific terms such as “Carcinoma, Pancreatic Ductal” [Mesh], “Pancreatic Neoplasms” [Mesh], “Cholangiocarcinoma” [Mesh], “Gallbladder Neoplasms” [Mesh], “Pancreatic Intraductal Neoplasms” [Mesh], “Bile Duct Neoplasms” [Mesh], “Liver Neoplasms” [Mesh], “Carcinoma, Hepatocellular” [Mesh], “Biliary Tract Neoplasms” [Mesh], “Bone Density” [Mesh], “Osteosarcopenia” [Title/Abstract], “Osteopenia” [Title/Abstract], among others. This search was limited to studies conducted in the English language involving human subjects. For a more detailed insight into our search strategy, we refer readers to **Supplementary Material 1**. Furthermore, we conducted supplementary searches in grey literature using Google Scholar and performed a manual examination of the reference lists from qualifying studies. Following the protocols established by the Cochrane collaboration, the results from both manual and electronic sources were consolidated using the Covidence software to facilitate effective data management.

### 2.2 Inclusion and exclusion criteria

We have defined a predefined set of inclusion and exclusion criteria for the selection of articles. The inclusion criteria encompassed the following aspects: (i) Inclusion of articles centered on patients with a diagnosis of HBPC. (ii) Investigation of the prognostic value of the baseline of preoperative osteopenia and osteosarcopenia. (iii) The presence of at least one among the designated endpoints, including overall survival (OS), recurrence-free survival (RFS), or postoperative major complications (Clavien-Dindo grade ≥ III). Conversely, exclusion criteria consisted of: (i) Research of alternative typologies, such as animal studies, reviews, case reports, or conference abstracts. (ii) Research projects wherein the essential data required for hazard ratio (HR) or odds ratio (OR) computation regarding the designated endpoints was notably absent from both the textual content and the publicly accessible records. In cases where multiple studies contained overlapping cohorts of patients, priority was given to articles that presented comprehensive data and adhered to rigorous methodological standards (16).

### 2.3 Data extraction and quality assessment

During the process of data extraction, we systematically and comprehensively gathered essential information. This encompassed a wide range of details, including the author, publication year, study characteristics (region, period, and design), treatment, cancer type, demographic characteristics (such as sample size and gender distribution), outcome, the assessment of method and site, and the cut-off points. HR, OR, and their respective 95% confidence intervals (CIs) are predominantly sourced from multivariate analysis. In situations where such statistical values were not available, we either turned to univariate analysis or employed the Engauge Digitizer software to extract data from survival analysis graphs. In evaluating the caliber of the observational studies, we employed the Newcastle-Ottawa Scale (NOS) score. Studies that achieved a score of six points or higher were considered to be of high quality. It is worth highlighting that each stage of this procedure, encompassing literature retrieval, filtration, data acquisition, and evaluation of quality, underwent comprehensive and autonomous execution by a trio of investigators. In cases of disparities or contentions, such issues were referred to the senior author for adjudication.

### 2.4 Statistical methods

In this study, Stata 15.0 software was utilized for the purpose of statistical analysis. The data presentation was executed using forest plots. To gauge the presence of heterogeneity within the dataset, we employed both Cochran’s Q test and I^2^ statistics. Either “*P*-value < 0.1” or “I^2^ value > 50%” was considered statistically significant heterogeneity. In cases of significant heterogeneity, we adopted a random-effects model, employing the DerSimonian-Laird method. Conversely, in the absence of significant heterogeneity, we adopted a fixed-effect model with the Inverse Variance method. We also conducted an assessment of potential publication bias, utilizing both Begg’s test (17) and Egger’s test (18). In order to evaluate the robustness of our observations, a sensitivity analysis was systematically executed, entailing the methodical exclusion of each individual study. Furthermore, we conducted subgroup analyses, taking into account factors such as Cox regression analysis, cancer type, and cut-off.

## 3 Results

### 3.1 Literature assessment and selective inclusion of studies

**Figure 1:**
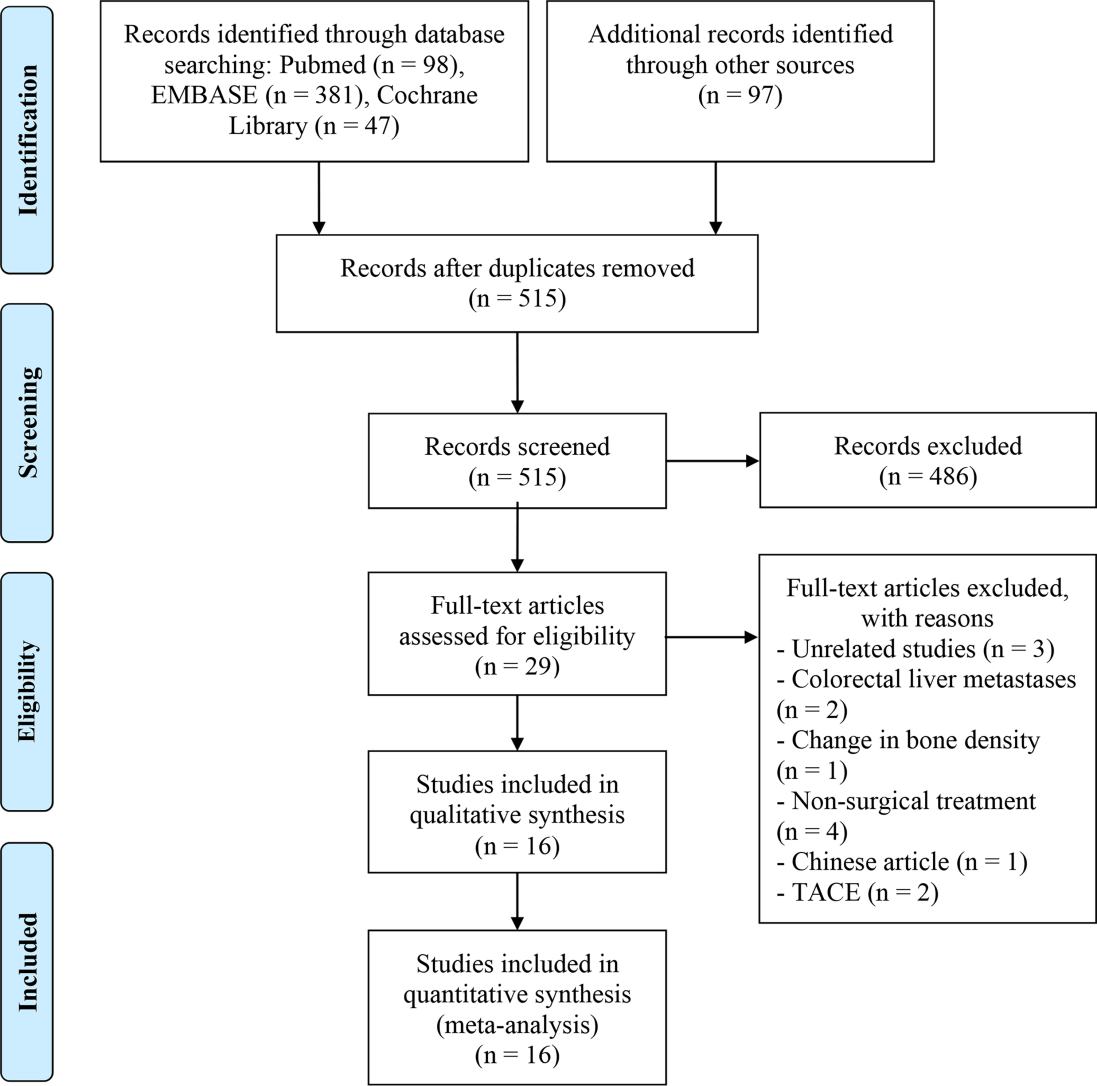
The flow diagram of identifying eligible studies.

**Figure 1** visually presents the results, as shown in the PRISMA flow diagram. In the preliminary stage, a cumulative sum of 623 research papers was discerned by means of database queries in conjunction with manual exploration. After removing duplicate entries, 515 distinct articles remained. A thorough review of the titles and abstracts led to the exclusion of 486 articles that did not meet the eligibility criteria. From the remaining pool, 29 articles underwent a comprehensive full-text review, resulting in the inclusion of 16 studies that met the established criteria for analysis (8, 19–35).

### 3.2 Study characteristics

**Table 1** provides an overview of the key characteristics of the studies included in this analysis. A total of 2,599 patients were enrolled in the research, with 65.81% being male. Geographically, thirteen studies were conducted in Japan, two in USA, and one in the Germany. These studies exhibited diversity in terms of the specific types of HBPC they enrolled. Five studies focused on patients with hepatocellular carcinoma (HCC), six on BTC, and five on PC. Fifteen studies used CT scans to measure BMD at the 11th thoracic vertebra to diagnose osteoporosis. Six studies defined osteopenia as an actual BMD lower than the calculated standardized BMD (308.82-2.49×age in men and 311.84-2.41×age in women). In five studies, patients with BMD < 160 HU were considered to have osteopenia. Furthermore, the 16 cohorts received NOS scores ranging from 6 to 8 (**Table 1**).

**Table 1:** Main characteristics of the studies included.

Study	Study design	Study period	Study region	Treatment	Cancer Type	Sample size	Gender (male/female)	Outcome	Cut-off	Method and site
Miki et al. (30)	R	07/2008-06/2022	Japan	Hepatectomy	BTC	71	46/25	Osteopenia (OS, RFS)	160 HU	CT, Th11
Watanabe et al. (28)	R	08/2007-08/2021	Japan	Biliary confluence resection combined with major hepatectomy	BTC	58	42/16	Osteopenia (OS, PCs)	160 HU	CT, Th11
Meister et al. 2023	R	05/2008-12/2019	Germany	Hepatectomy	HCC	100	72/28	Osteopenia (OS, RFS, PCs)	160/175 HU^a^	CT, Th11
Cameron et al. (26)	R	10/2011-01/2018	USA	Surgical resection	PC	209	120/89	Osteopenia (OS)	145 HU	CT, (Lh1-5)/5
Tamura et al (25).	R	09/2002-12/2017	Japan	Pancreatoduodenectomy	BTC	111	86/25	Osteopenia (OS, RFS)	75/74 HU^a^	CT, Th11
Abe K et al (24).	R	01/2014-12/2018	Japan	Pancreaticoduodenectomy or distal pancreatectomy	PC	56	30/26	Osteopenia (OS, RFS, PCs)	160 HU	CT, Th11
Toshima et al (23).	R	01/1998-12/2015	Japan	Liver transplantation	HCC	193	109/84	Osteopenia (OS)	Age-adjusted^b^	CT, Th11
Sharshar et al (22).	R	10/2003-02/2016	Japan	Surgical resection	PC	275	154/121	Osteopenia (OS, RFS, PCs)	138/129 HU^a^	CT, Th11
Motomura et al. (21),	R	03/2009-01/2019	Japan	Surgical resection	PC	91	48/43	Osteopenia (OS, RFS)	Age-adjusted^b^	CT, Th11
Yao et al. (8)	R	2005-2015	Japan	Surgical resection	BTC	181	98/83	Osteopenia (OS, RFS, PCs)	169 HU	CT, Th11
Miyachi et al (20).	R	04/2005-03/2015	Japan	Hepatectomy	HCC	465	367/98	Osteopenia (OS, PCs)	160 HU	CT, Th11
Sharma et al (19).	R	02/2002-12/2013	USA	Liver transplantation	HCC	118	92/26	Osteopenia (OS)	160 HU	CT, Th11
Abe T et al. 2021	R	01/2012-12/2018	Japan	Surgical resection	PC	265	151/114	Osteopenia, Osteo-sarcopenia (OS, RFS)	Age-adjusted^b^; 47.1/36.6 cm^2^/m^2c^	CT, Th11; L3
Matsumoto et al. 2020	R	07/2007-12/2018	Japan	Surgical resection	BTC	138	94/44	Osteopenia, Osteo-sarcopenia (OS, RFS)	Age-adjusted^b^; 26.4/13.3 cm^2d^	CT, Th11; L3
Taniai et al. (34)	R	01/2007-12/2019	Japan	Hepatectomy	BTC	41	21/20	Osteopenia, Osteo-sarcopenia (OS, RFS)	Age-adjusted^b^; 31.5/14.9 cm^2d^	CT, Th11; L3
Yanagaki et al. (35)	R	01/2001-12/2018	Japan	Hepatectomy	HCC	227	46/181	Osteopenia, Osteo-sarcopenia (OS, RFS)	Age-adjusted^b^; 11.0/7.4 cm^2^/m^2e^	CT, Th11; L3

^a^male/female; ^b^Osteopenia was defined as the actual bone mineral density (BMD) below the calculated standard BMD (men: 308.82-2.49 × age (year) and women: 311.84-2.41 × age (year)); ^c^SMI (cm2/m2) was calculated as skeletal muscle area (cm2) divided by the square of the height (m2) (male/female); ^d^PMA was calculated as the major axis × the minor axis × π (male/female); ^e^Skeletal muscle index (SMI) was calculated as the psoas muscle area divided by height in meters squared (male/female).

R, retrospective study; TACE, transarterial chemoembolization; HCC, hepatocellular carcinoma; BTC, biliary tract cancer; PC, pancreatic cancer; PCs, postoperative complications (Clavien–Dindo grade ≥ III); OS, overall survival; RFS, relapse-free survival; HU, Hounsfield units; Th11, 11th thoracic vertebra; Lh3, 3th lumbar vertebra; Lh1-5, 1-5th lumbar vertebra; CT, computed tomography; L3, third lumbar vertebra.

### 3.3 Association of osteopenia with overall survival

**Figure 2:**
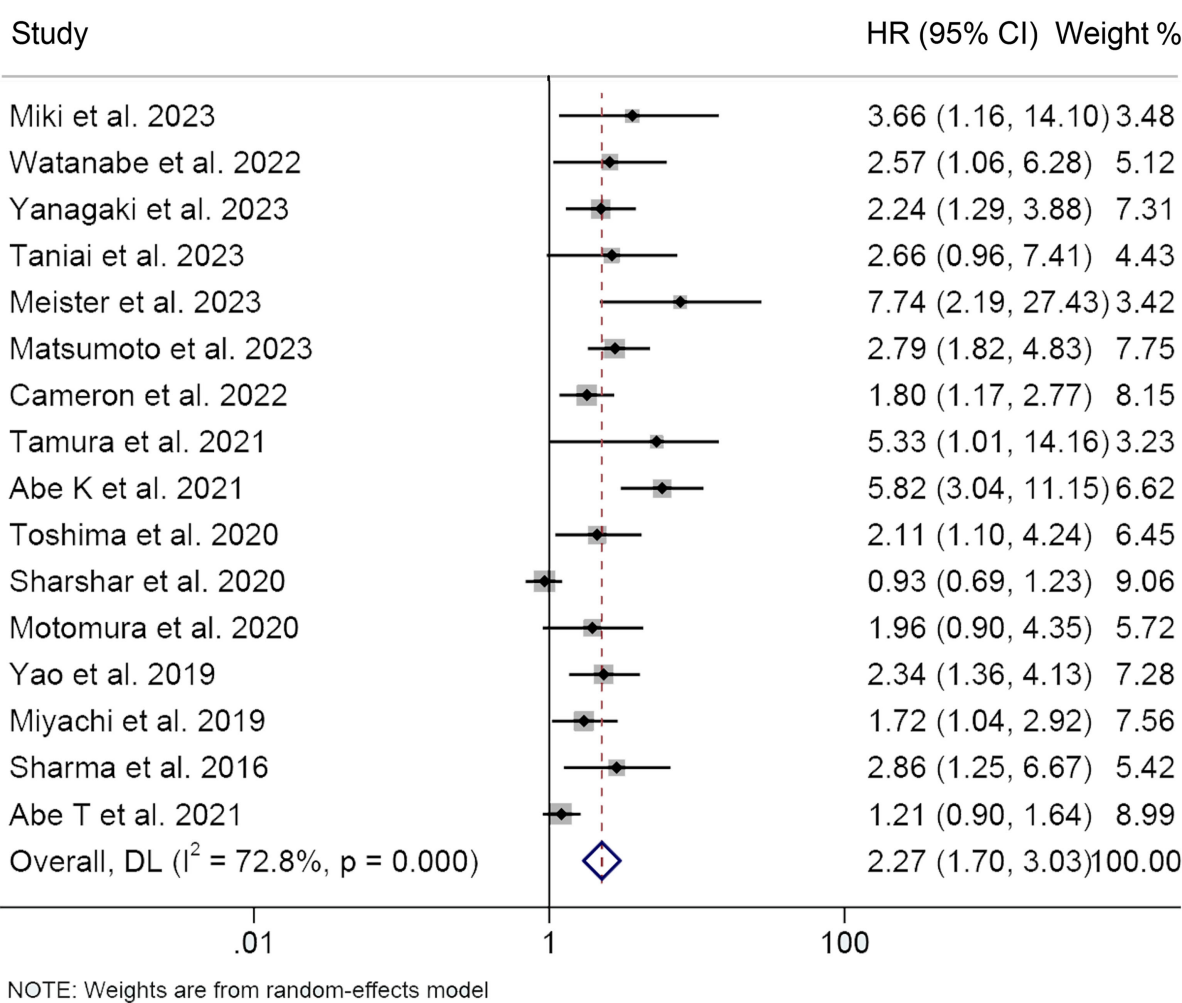
Forest plots of the relationship between osteopenia and overall survival. HR, hazard ratio; CI, confidence interval.

In total, 16 studies investigated the impact of preoperative osteopenia on the OS of patients with HBPC, encompassing 2,599 individuals. We applied a random-effects model due to significant heterogeneity, as indicated by Cochran’s Q test and I^2^ statistics (I^2^ = 72.8%, *p* < 0.001). Our study revealed that individuals diagnosed with osteopenia exhibited significantly worse OS (HR: 2.27, 95% CI: 1.70-3.03, *p* < 0.001, **Figure 2**) in contrast to those devoid of osteopenia.

We performed subgroup analysis based on Cox regression analysis, cancer type, and cut-off values (**Table 2**). Both univariate and multivariate analyses consistently supported that osteoporosis predicted shorter OS (multivariate analyses, HR: 2.46, 95% CI: 1.79-3.37, *p* < 0.001; univariate analyses, HR: 2.06, 95% CI: 1.40-2.80, *p* < 0.001), reaffirming the robustness of our research findings. Subgroup examination, stratified by cancer classifications, demonstrated that for individuals afflicted with HCC (HR: 2.31, 95% CI: 1.63-3.25, *p* < 0.001), BTC (HR: 2.75, 95% CI: 2.03-3.72, *p* < 0.001), and PC (HR: 1.76, 95% CI: 1.05-2.95, *p* = 0.031), the presence of osteopenia was associated with markedly diminished OS (**Table 2**). Furthermore, it is noteworthy that the adverse impact on overall survival attributed to osteopenia persisted, irrespective of whether the diagnostic criterion applied was a 160 HU cut-off or age-adjusted BMD.

**Table 2:** Subgroup analysis of the association between osteopenia and the outcomes for hepatobiliary-pancreatic cancers.

Variable	Included studies	Test of association			Test of heterogeneity		
		HR	95% CI	*p*-value	Modal	I^2^	*p*-value
Overall survival							
Cox regression analysis							
Multivariate analysis	7	2.46	1.79-3.37	*p* < 0.001	R	13.1%	*p* = 0.329
Univariate analysis	9	2.06	1.40-2.80	*p* < 0.001	R	80.8%	*p* < 0.001
Cancer type							
Hepatocellular carcinoma	5	2.31	1.63-3.25	*p* < 0.001	R	21.0%	*p* = 0.281
Biliary tract cancer	6	2.75	2.03-3.72	*p* < 0.001	R	0	*p* = 0.911
Pancreatic cancer	5	1.76	1.05-2.95	*p* = 0.031	R	86.2%	*p* < 0.001
Cut-off							
Age-adjusted	6	1.97	1.39-2.79	*p* < 0.001	R	54.9%	*p* = 0.050
160 HU	5	2.97	1.79-4.92	*p* < 0.001	R	53.0%	*p* = 0.075
Other	5	2.24	1.19-4.21	*p* = 0.013	R	82.8%	*p* < 0.001
Recurrence-free survival							
Cox regression analysis							
Multivariate analysis	5	2.64	1.87-3.72	*p* < 0.001	R	0	*p* = 0.603
Univariate analysis	6	1.68	1.14-2.48	*p* = 0.009	R	81.3%	*p* < 0.001
Cancer type							
Hepatocellular carcinoma	2	1.72	1.21-2.45	*p* = 0.003	R	0	*p* = 0.653
Biliary tract cancer	5	2.61	1.93-3.51	*p* < 0.001	R	0	*p* = 0.618
Pancreatic cancer	4	1.56	0.93-2.62	*p* = 0.090	R	84.8%	*p* < 0.001
Cut-off							
Age-adjusted	5	1.73	1.29-2.32	*p* < 0.001	R	49.4%	*p* = 0.095
160 HU	2	2.73	1.45-5.13	*p* = 0.002	R	23.8%	*p* = 0.252
Other	4	2.09	0.88-4.98	*p* = 0.095	R	87.3%	*p* < 0.001

HR, hazard ratio; CL, confidence interval; R, random-effect model; F, fixed-effect model; HU, hounsfield units.

### 3.4 Preoperative osteopenia and recurrence-free survival

**Figure 3:**
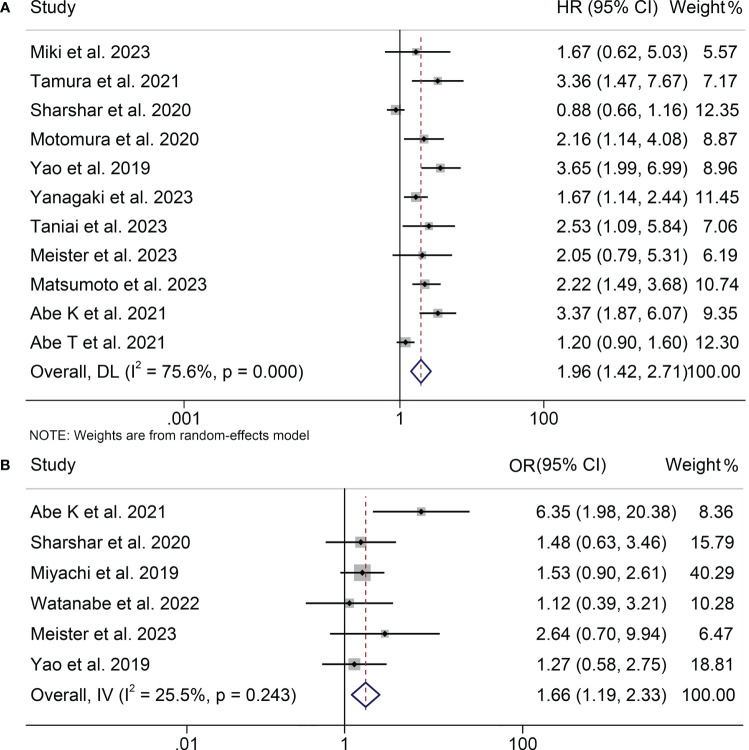
Forest plots of the relationship between osteopenia and recurrence-free survival **(A)** and postoperative major complications **(B)**. HR, hazard ratio; OR, odds ratio; CI, confidence interval.

Eleven studies, involving 1556 patients, examined the relationship between osteopenia and RFS in HBPC patients. Due to significant heterogeneity among the studies (I^2^ = 75.6%, *p* < 0.001), a random-effects model was employed. The pooled analysis revealed a markedly shortened RFS in HPBC patients suffering from osteopenia (HR: 1.96, 95% CI: 1.42-2.71, *p* < 0.001, **Figure 3A**).

The results of both univariate and multivariate analyses substantiate the reliability of our research findings, which consistently supported the prediction of shorter RFS by osteopenia (multivariate analyses, HR: 2.64, 95% CI: 1.87-3.72, *p* < 0.001; univariate analyses, HR: 1.68, 95% CI: 1.14-2.48, *p* = 0.009; **Table 2**). Subgroup analysis by cancer type indicated that osteopenia significantly predicted worse RFS in patients with HCC (HR: 1.72, 95% CI: 1.21-2.45, *p* = 0.003) and BTC (HR: 2.61, 95% CI: 1.93-3.51, *p* < 0.001; **Table 2**). Nevertheless, in the case of PC, a tendency towards reduced RFS was observed among osteopenia patients; however, this trend did not attain statistical significance (HR: 1.56, 95% CI: 0.93-2.62, *p* = 0.090; **Table 2**). Considering that only four PC studies were included, a larger number of studies are needed to further confirm the relationship between osteopenia and the prognosis of patients treated with surgery for PC. Moreover, irrespective of the criteria employed to diagnose osteopenia, be it through the application of a threshold of 160 HU or the utilization of age-adjusted BMD, a consistent and robust correlation was observed with a poorer RFS outcome.

### 3.5 Baseline osteopenia and postoperative major complications

This analysis, depicted in **Figure 3B**, assesses the influence of osteopenia on the incidence of major postoperative complications. A low level of heterogeneity was observed, as indicated by the I^2^ value (I^2^ = 25.5, *p* = 0.243), prompting the utilization of a fixed-effects model. The results demonstrated a pooled OR of 1.66 (95% CI: 1.19-2.33) based on six studies encompassing 1135 patients. This signifies a statistically significant elevation in the risk of major postoperative complications among individuals with osteopenia in comparison to those without this condition.

### 3.6 Sensitivity analysis and publication bias test for osteopenia and related outcomes

**Figure 4:**
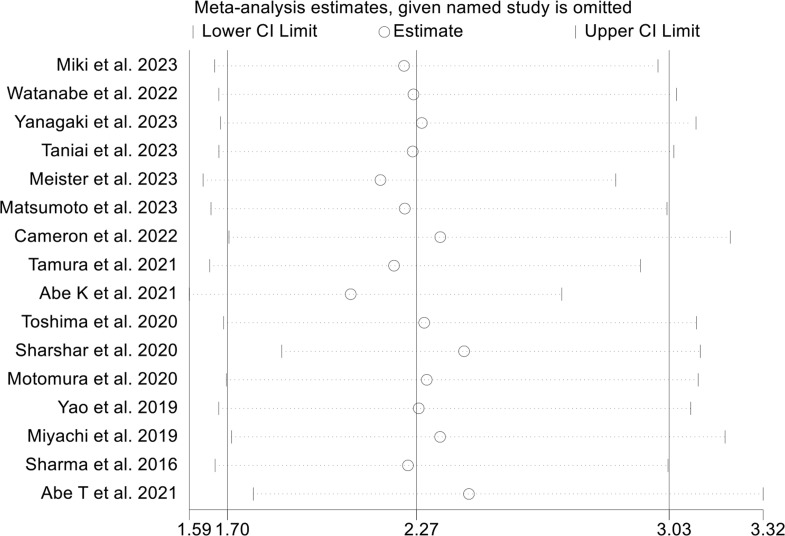
Sensitivity analysis of the association between osteopenia and overall survival. CI, confidence interval.

**Figure 5:**
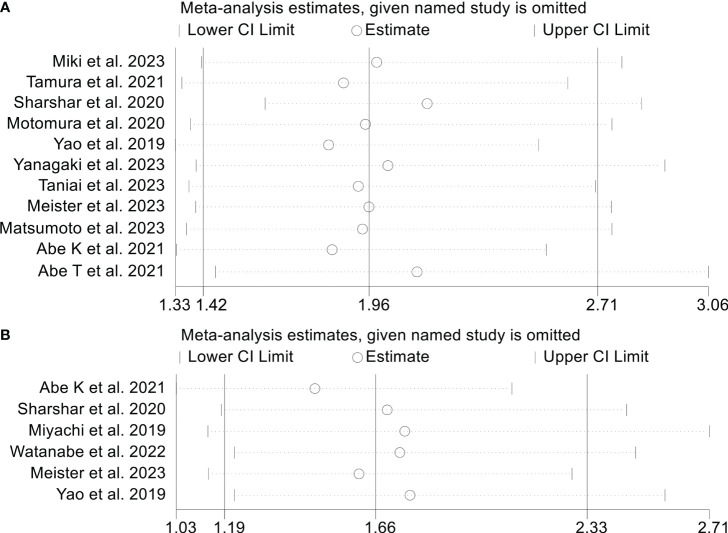
Sensitivity analysis of the association between osteopenia and recurrence-free survival **(A)** and postoperative major complications **(B)**. CI, confidence interval.

Sensitivity analysis and investigations of publication bias were conducted to assess the robustness of the association between osteopenia and survival outcomes. The exclusion of individual studies did not exert a significant impact on pooled HR for OS, which ranged from 2.07 (95% CI: 1.59-2.71, post-exclusion of Abe K et al., 2021) to 2.43 (95% CI: 1.78-3.32, post-exclusion of Abe T et al., 2021, **Figure 4**). Similarly, for RFS, the HR exhibited minimal variations, ranging from 1.83 (95% CI: 1.33-2.51, post-exclusion of Yao et al., 2019) to 2.12 (95% CI: 1.46-3.06, after excluding Abe T et al., 2021, **Figure 5A**).

The possibility of publication bias was assessed using Begg’s and Egger’s tests. Notably, the results of these tests did not indicate any significant publication bias for RFS (Egger’s test: *p* = 0.533, Begg’s test: *p* = 0.108). However, the funnel plot, Begg’s and Egger’s tests confirmed a significant publication bias in the case of osteopenia and OS (Egger’s test: *p* = 0.001, Begg’s test: *p* = 0.017, **Supplementary Figure S1A**). To address this issue, we employed the trim and fill method, a valuable tool for estimating the number of potentially missing studies in OS. The results of this analysis indicated that even after incorporating these potentially missing studies, there was no discernible alteration in the pooled HR for OS (**Supplementary Figure S1B**).

In a similar vein, sensitivity analysis and assessment of publication bias have been employed to investigate the stability and reliability of the connection between osteopenia and postoperative major complications. The OR of the primary analysis remained unaltered upon the exclusion of any individual study (**Figure 5B**). Rigorous scrutiny through Egger’s and Begg’s tests affirmed the absence of notable publication bias (Egger’s test: *p* = 0.325, Begg’s test: *p* = 0.452).

### 3.7 Relationship of osteosarcopenia with overall and recurrence-free survival

**Figure 6:**
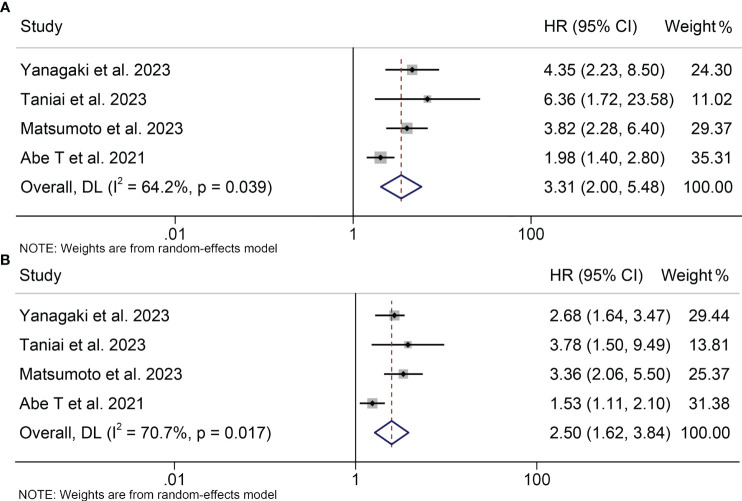
Forest plots of the relationship between osteosarcopenia and overall survival **(A)** and recurrence-free survival **(B)**. HR, hazard ratio; CI, confidence interval.

A total of four studies, involving 671 patients, were included in this analysis, examining the impact of preoperative osteosarcopenia on OS or RFS in HBPC patients. Notably, there was significant heterogeneity among the included studies (OS: I^2^ = 64.2%, *p* = 0.039; RFS: I^2^ = 70.7%, *p* = 0.017), so a random-effects model was employed. Our findings demonstrated that patients with osteosarcopenia had significantly inferior OS (HR: 3.31, 95% CI: 2.00-5.48, *p* < 0.001, **Figure 6A**) and RFS (HR: 2.50, 95% CI: 1.62-3.84, *p* < 0.001, **Figure 6B**) compared to those without osteosarcopenia.

Sensitivity analyses and publication bias tests for the correlation between osteosarcopenia and OS as well as RFS revealed that the exclusion of any single study did not exert a substantial impact on the pooled results, and no indications of publication bias were detected (**Supplementary Figure S2**). As elucidated above, our findings exhibit robustness and reliability.

## 4 Discussion

In this investigation, we have gathered evidence from 18 studies indicating a significant correlation between osteopenia and osteosarcopenia with lower OS and RFS in surgically treated HBPC patients. Furthermore, patients afflicted with osteopenia displayed a considerably elevated incidence of postoperative complications.

The etiology of osteopenia in cancer patients is complicated, encompassing age-related factors alongside osteoporotic contributors induced by cancer treatments (36). Inflammation serves as a hallmark of cancer, fostering accelerated osteopenia (37). Several elements, including prostaglandin E2 and cyclooxygenase, have been identified as contributors to cancer-related inflammation, which accelerates the degradation of bone density (38). Lifestyle factors commonly observed in malignancy, such as fatigue, sedentary behavior, inadequate calcium intake, weight loss, fragility, and malnutrition, are known to exacerbate rapid bone loss in cancer patients. Additionally, reduced mechanical loading, which is often caused by cancer-induced fatigue and sedentary behavior, also contributes to the development of osteopenia (39, 40).

Pereira et al. demonstrated a substantial correlation between pre-sarcopenia, as defined by the European Working Group on Sarcopenia in Older People (41), and abnormal BMD (11). Osteopenia is regarded as the initial indicator within the spectrum known as bone loss-sarcopenia-frailty (19, 20). As previously elucidated, sarcopenia has been associated with an adverse prognosis in patients following hepatobiliary and pancreatic surgery (13–15). Our study affirms that the prognosis of surgical patients can be determined by assessing osteopenia before the development of sarcopenia.

The reasons for the adverse postoperative outcomes observed in patients with HBPC suffering from osteopenia remain obscure. Osteopenia and sarcopenia represent inherent components of the aging trajectory, rendering individuals more susceptible to falls, fractures, and overall frailty, particularly prevalent in the elderly population (42). One inevitable outcome of the aging process is the emergence of weakness. In clinical practice, frailty is used to categorize cancer patients who are at risk for unfavorable prognoses, as well as to identify those individuals who may experience early morbidity and death following cancer treatment (43).

The influence of osteopenia on prognostic outcomes may be intricately linked to immune function. Recent research has probed the potential interaction between the skeletal system and the immune system, commonly referred to as “osteoimmunology” (20). In the realm of sarcopenia, NF-κB stands out as a pivotal molecular actor (44). The ligand for the receptor activator of NF-κB (RANK) initiates the process of osteoclastogenesis, ultimately culminating in osteopenia, with osteoprotegerin acting as its inhibitor (45). Remarkably, RANK is also expressed in the skeletal muscle. In this scenario, activation of the NF-κB pathway predominantly impedes myogenic differentiation, thereby leading to skeletal muscle dysfunction or degeneration, which is one of the hallmarks of sarcopenia (46, 47). Furthermore, NF-κB also promotes cancer cell migration and invasion by upregulating Snail expression, subsequently suppressing E-cadherin (48).

Our investigation further demonstrates an association between osteosarcopenia and an unfavorable prognosis in HBPC patients undergoing surgery. CD109, an inositol-anchored glycoprotein expressed in numerous cancers, emerges as one of the regulatory factors governing bone homeostasis (49). CD109 is implicated in promoting cancer cell proliferation (50) and metastasis through the JAK/STAT signaling pathway (51). Simultaneously, CD109 also plays a role in regulating the activity of osteoclasts and osteoblasts (49). Evidence indicates that the expression of microRNA-188 modulates histone deacetylase 9 and mTOR complex 2, mTOR-independently associated regulatory proteins, which may manifest as increased fat accumulation within the bone marrow and diminished bone formation (51). Ultimately, this fat accumulation contributes to osteopenia (51). Sarcopenia is also associated with the levels of interleukin (IL)-23 (52) and tumor necrosis factor (TNF)-α (53). Dendritic cell-produced IL-23 also activates T helper 17 cells, which aids in the development of tumors (54). TNF-α activates the signal transducer and activator of transcription 3 and NF-kB, promoting cancer growth (55). Studies indicate that these variables influence the development of tumors and sarcopenia. Osteosarcopenia may be influenced by the progression of cancer, where cholestasis induced by biliary malignancy reduces the absorption of vitamin K and causes bone resorption (56). As previously delineated, the molecular processes linking osteopenia and sarcopenia to cancer are different. Osteosarcopenia represents a confluence of osteopenia and sarcopenia, potentially heralding the advanced debility of cancer patients. We reveal that preoperative osteopenia and osteosarcopenia have the potential to lead to poorer OS and RFS in HBPC patients undergoing surgery. Therefore, clinicians should assess bone density and intervene to maximize the benefit to patients when conducting surgical treatment. Our findings highlight the importance of preoperative interventions aimed at preserving bone density, which have the potential to improve patient prognosis.

Currently, several therapeutic approaches, including exercise and anti-osteoporotic pharmaceuticals (denosumab, teriparatide, and bisphosphonates), branched-chain amino acids, vitamin D, calcium, and symbiotics, are available to enhance bone mass and prevent fractures in patients with osteoporosis (57–59). Although it may take time for these interventions to result in BMD, reversing the preoperative osteopenia condition may help to improve the postoperative prognosis.

The present analysis exhibits certain constraints. Firstly, the number of studies concerning osteosarcopenia is limited, and there is an insufficient volume of research dedicated to exploring the impact of osteosarcopenia on major postoperative complications. Secondly, there was some heterogeneity among the included studies, and the diagnostic criteria for osteopenia were not completely consistent. However, our publication bias test and sensitivity analysis proved that the results were stable and reliable. Finally, the cut-off values for the same diagnostic metric differed among distinct studies. Hence, to attain more reliable conclusions, there is an urgent requirement for a worldwide, multicenter investigation to explore the impact of osteopenia on HBPC patients’ prognosis.

## 5 Conclusion

In summary, preoperative osteopenia and osteosarcopenia have the potential to forecast poorer OS and RFS for HBPC patients undergoing surgery. Our findings underscore the importance of preoperative interventions aimed at preserving bone density, which could potentially improve patient prognosis.

## Data availability statement

The original contributions presented in the study are included in the article/**Supplementary Material**. Further inquiries can be directed to the corresponding author.

## Author contributions

HJ: Conceptualization, Methodology, Project administration, Resources, Supervision, Validation, Writing – review & editing. XW: Conceptualization, Data curation, Methodology, Project administration, Software, Supervision, Visualization, Writing – original draft. MW: Conceptualization, Methodology, Writing – original draft. QL: Methodology, Writing – original draft. WH: Formal analysis, Methodology, Writing – original draft. YT: Investigation, Resources, Writing – original draft. YZ: Data curation, Writing – original draft. CL: Investigation, Writing – original draft. YL: Methodology, Writing – original draft. AY: Supervision, Validation, Writing – original draft.

## Supplementary material

Supplementary Figure 1
**(A)** Funnel plots of the relationship between osteopenia and overall survival; These two diagonal lines are the confidence intervals for the funnel plot; the vertical line in the middle represents the combined HR values, and when unbiased (ideally), the individual studies should be equally distributed on both sides of the vertical line in an inverted funnel shape. **(B)** The picture of the trim-and-fill method. Theta, the effect estimate; Se_theta, the corresponding standard error; The circles represent the studies included in this meta-analysis; Boxes with circles represent additional studies of the trim and fill method.

Supplementary Figure 2 Sensitivity analysis of the association between osteosarcopenia and overall survival **(A)** and recurrence-free survival **(B)**. CI, confidence interval.

## References

[1] Cazacu IM Singh BS Saftoiu A Bhutani MS. Recent developments in hepatopancreatobiliary EUS. Endosc Ultrasound. (2019) 8:146–50. doi: 10.4103/eus.eus_20_19 PMC6590003 31031329

[2] Kovacevic B Karstensen JG Havre RF Pham KD Giovannini M Dabizzi E. Initial experience with EUS-guided microbiopsy forceps in diagnosing pancreatic cystic lesions: A multicenter feasibility study (with video). Endosc Ultrasound. (2018) 7:383–8. doi: 10.4103/eus.eus_16_18 PMC6289018 30168479

[3] Sãftoiu A Bhutani MS Itoi T Arcidiacono PG Bories E Cazacu IM. Changes in tumor vascularity depicted by contrast-enhanced EUS as a predictor of prognosis and treatment efficacy in patients with unresectable pancreatic cancer (PEACE): A study protocol. Endosc Ultrasound. (2019) 8:235–40. doi: 10.4103/eus.eus_16_19 PMC6714481 31249159

[4] Bray F Ferlay J Soerjomataram I Siegel RL Torre LA Jemal A. Global cancer statistics 2018: GLOBOCAN estimates of incidence and mortality worldwide for 36 cancers in 185 countries. CA Cancer J Clin. (2018) 68:394–424. doi: 10.3322/caac.21492 30207593

[5] Peery AF Crockett SD Murphy CC Lund JL Dellon ES Williams JL. Burden and cost of gastrointestinal, liver, and pancreatic diseases in the United States: update 2018. Gastroenterology. (2019) 156:254–72.e211. doi: 10.1053/j.gastro.2018.08.063 30315778 PMC6689327

[6] Blumenthal GM Pazdur R. Approvals in 2016: the march of the checkpoint inhibitors. Nat Rev Clin Oncol. (2017) 14:131–2. doi: 10.1038/nrclinonc.2017.15 28218255

[7] Banales JM Marin JJG Lamarca A Rodrigues PM Khan SA Roberts LR. Cholangiocarcinoma 2020: the next horizon in mechanisms and management. Nat Rev Gastroenterol Hepatol. (2020) 17:557–88. doi: 10.1038/s41575-020-0310-z PMC7447603 32606456

[8] Yao S Kaido T Okumura S Iwamura S Miyachi Y Shirai H. Bone mineral density correlates with survival after resection of extrahepatic biliary Malignancies. Clin Nutr. (2019) 38:2770–7. doi: 10.1016/j.clnu.2018.12.004 30595376

[9] Pickhardt PJ Pooler BD Lauder T del Rio AM Bruce RJ Binkley N. Opportunistic screening for osteoporosis using abdominal computed tomography scans obtained for other indications. Ann Intern Med. (2013) 158:588–95. doi: 10.7326/0003-4819-158-8-201304160-00003 PMC3736840 23588747

[10] Edwards BJ Sun M Zhang X Holmes HM Song J Khalil P. Fractures frequently occur in older cancer patients: the MD Anderson Cancer Center experience. Support Care Cancer. (2018) 26:1561–8. doi: 10.1007/s00520-017-3962-7 29197959

[11] Pereira FB Leite AF de Paula AP. Relationship between pre-sarcopenia, sarcopenia and bone mineral density in elderly men. Arch Endocrinol Metab. (2015) 59:59–65. doi: 10.1590/2359-3997000000011 25926116

[12] Inoue T Maeda K Nagano A Shimizu A Ueshima J Murotani K. Related factors and clinical outcomes of osteosarcopenia: A narrative review. Nutrients. (2021) 13(2):291. doi: 10.3390/nu13020291 33498519 PMC7909576

[13] Wang P Wang S Ma Y Li H Liu Z Lin G. Sarcopenic obesity and therapeutic outcomes in gastrointestinal surgical oncology: A meta-analysis. Front Nutr. (2022) 9:921817. doi: 10.3389/fnut.2022.921817 35938099 PMC9355157

[14] Cao Q Xiong Y Zhong Z Ye Q. Computed tomography-assessed sarcopenia indexes predict major complications following surgery for hepatopancreatobiliary Malignancy: A meta-analysis. Ann Nutr Metab. (2019) 74:24–34. doi: 10.1159/000494887 30513518

[15] Ratnayake CB Loveday BP Shrikhande SV Windsor JA Pandanaboyana S. Impact of preoperative sarcopenia on postoperative outcomes following pancreatic resection: A systematic review and meta-analysis. Pancreatology. (2018) 18:996–1004. doi: 10.1016/j.pan.2018.09.011 30287167

[16] Zhang L Kuang T Chai D Deng W Wang P Wang W. The use of antibiotics during immune checkpoint inhibitor treatment is associated with lower survival in advanced esophagogastric cancer. Int Immunopharmacol. (2023) 119:110200. doi: 10.1016/j.intimp.2023.110200 37099942

[17] Egger M Davey Smith G Schneider M Minder C. Bias in meta-analysis detected by a simple, graphical test. Bmj. (1997) 315:629–34. doi: 10.1136/bmj.315.7109.629 PMC2127453 9310563

[18] Begg CB Mazumdar M. Operating characteristics of a rank correlation test for publication bias. Biometrics. (1994) 50:1088–101. doi: 10.2307/2533446 7786990

[19] Sharma P Parikh ND Yu J Barman P Derstine BA Sonnenday CJ. Bone mineral density predicts posttransplant survival among hepatocellular carcinoma liver transplant recipients. Liver Transpl. (2016) 22:1092–8. doi: 10.1002/lt.24458 PMC4961525 27064263

[20] Miyachi Y Kaido T Yao S Shirai H Kobayashi A Hamaguchi Y. Bone mineral density as a risk factor for patients undergoing surgery for hepatocellular carcinoma. World J Surg. (2019) 43:920–8. doi: 10.1007/s00268-018-4861-x 30465085

[21] Motomura T Uchiyama H Iguchi T Ninomiya M Yoshida R Honboh T. Impact of osteopenia on oncologic outcomes after curative resection for pancreatic cancer. In Vivo. (2020) 34:3551–7. doi: 10.21873/invivo.12198 PMC7811648 33144467

[22] Sharshar M Kaido T Shirai H Okumura S Yao S Miyachi Y. Impact of the preoperative bone mineral density on the outcomes after resection of pancreatic cancer. Surg Today. (2020) 50:757–66. doi: 10.1007/s00595-019-01954-y 31925578

[23] Toshima T Yoshizumi T Kosai-Fujimoto Y Inokuchi S Yoshiya S Takeishi K. Prognostic impact of osteopenia in patients who underwent living donor liver transplantation for hepatocellular carcinoma. World J Surg. (2020) 44:258–67. doi: 10.1007/s00268-019-05206-5 31624895

[24] Abe K Furukawa K Okamoto T Matsumoto M Futagawa Y Haruki K. Impact of osteopenia on surgical and oncological outcomes in patients with pancreatic cancer. Int J Clin Oncol. (2021) 26:1929–37. doi: 10.1007/s10147-021-01986-w 34232427

[25] Tamura S Ashida R Sugiura T Okamura Y Ito T Yamamoto Y. The prognostic impact of skeletal muscle status and bone mineral density for resected distal cholangiocarcinoma. Clin Nutr. (2021) 40:3552–8. doi: 10.1016/j.clnu.2020.12.011 33358552

[26] Cameron ME Underwood PW Williams IE George TJ Judge SM Yarrow JF. Osteopenia is associated with wasting in pancreatic adenocarcinoma and predicts survival after surgery. Cancer Med. (2022) 11:50–60. doi: 10.1002/cam4.4416 34791809 PMC8704155

[27] Meister FA Verhoeven S Mantas A Liu WJ Jiang D Heij L. Osteopenia is associated with inferior survival in patients undergoing partial hepatectomy for hepatocellular carcinoma. Sci Rep. (2022) 12:18316. doi: 10.1038/s41598-022-21652-z 36316524 PMC9622743

[28] Watanabe J Miki A Sakuma Y Shimodaira K Aoki Y Meguro Y. Preoperative osteopenia is associated with significantly shorter survival in patients with perihilar cholangiocarcinoma. Cancers (Basel). (2022) 14(9):2213. doi: 10.3390/cancers14092213 35565342 PMC9103099

[29] Loosen SH Jördens MS Schoon B Antoch G Luedde T Minko P. Sarcopenia indicate poor survival in patients undergoing transarterial chemoembolization (TACE) for hepatic Malignancies. J Cancer Res Clin Oncol. (2023) 149:6181–90. doi: 10.1007/s00432-022-04519-8 PMC10356883 36689060

[30] Miki A Sakuma Y Watanabe J Endo K Sasanuma H Teratani T. Osteopenia is associated with shorter survival in patients with intrahepatic cholangiocarcinoma. Curr Oncol. (2023) 30:1860–8. doi: 10.3390/curroncol30020144 PMC9955432 36826105

[31] Müller L Mähringer-Kunz A Auer TA Fehrenbach U Gebauer B Haubold J. Low bone mineral density is a prognostic factor for elderly patients with HCC undergoing TACE: results from a multicenter study. Eur Radiol. (2023) 33:1031–9. doi: 10.1007/s00330-022-09069-8 PMC9889510 35986768

[32] Abe T Nakata K Nakamura S Ideno N Ikenaga N Fujita N. Prognostic impact of preoperative osteosarcopenia for patients with pancreatic ductal adenocarcinoma after curative resection. Ann Surg Oncol. (2023) 30:6673–9. doi: 10.1245/s10434-023-13936-z 37466870

[33] Matsumoto M Onda S Igarashi Y Hamura R Uwagawa T Furukawa K. Osteosarcopenia is a significant predictor of recurrence and the prognosis after resection for extrahepatic bile duct cancer. Surg Today. (2023) 54 (5):407–18. doi: 10.1007/s00595-023-02747-0 37700170

[34] Taniai T Haruki K Yanagaki M Igarashi Y Furukawa K Onda S. Osteosarcopenia predicts poor prognosis for patients with intrahepatic cholangiocarcinoma after hepatic resection. Surg Today. (2023) 53:82–9. doi: 10.1007/s00595-022-02550-3 35831486

[35] Yanagaki M Haruki K Taniai T Igarashi Y Yasuda J Furukawa K. The significance of osteosarcopenia as a predictor of the long-term outcomes in hepatocellular carcinoma after hepatic resection. J Hepatobiliary Pancreat Sci. (2023) 30:453–61. doi: 10.1002/jhbp.1246 36181339

[36] Guise TA. Bone loss and fracture risk associated with cancer therapy. Oncologist. (2006) 11:1121–31. doi: 10.1634/theoncologist.11-10-1121 17110632

[37] Hanahan D Weinberg RA. The hallmarks of cancer. Cell. (2000) 100:57–70. doi: 10.1016/S0092-8674(00)81683-9 10647931

[38] Chen Z Maricic M Aragaki AK Mouton C Arendell L Lopez AM. Fracture risk increases after diagnosis of breast or other cancers in postmenopausal women: results from the Women's Health Initiative. Osteoporos Int. (2009) 20:527–36. doi: 10.1007/s00198-008-0721-0 PMC2895418 18766294

[39] Cabell L Pienkowski D Shapiro R Janura M. Effect of age and activity level on lower extremity gait dynamics: an introductory study. J Strength Cond Res. (2013) 27:1503–10. doi: 10.1519/JSC.0b013e318269f83d 22964857

[40] Clynes MA Edwards MH Buehring B Dennison EM Binkley N Cooper C. Definitions of sarcopenia: associations with previous falls and fracture in a population sample. Calcif Tissue Int. (2015) 97:445–52. doi: 10.1007/s00223-015-0044-z PMC4601152 26223791

[41] Cruz-Jentoft AJ Baeyens JP Bauer JM Boirie Y Cederholm T Landi F. Sarcopenia: European consensus on definition and diagnosis: Report of the European Working Group on Sarcopenia in Older People. Age Ageing. (2010) 39:412–23. doi: 10.1093/ageing/afq034 PMC2886201 20392703

[42] Takahashi K Nishikawa K Furukawa K Tanishima Y Ishikawa Y Kurogochi T. Prognostic significance of preoperative osteopenia in patients undergoing esophagectomy for esophageal cancer. World J Surg. (2021) 45:3119–28. doi: 10.1007/s00268-021-06199-w 34152448

[43] Guida JL Ahles TA Belsky D Campisi J Cohen HJ DeGregori J. Measuring aging and identifying aging phenotypes in cancer survivors. J Natl Cancer Inst. (2019) 111:1245–54. doi: 10.1093/jnci/djz136 PMC7962788 31321426

[44] Qiu J Thapaliya S Runkana A Yang Y Tsien C Mohan ML. Hyperammonemia in cirrhosis induces transcriptional regulation of myostatin by an NF-κB-mediated mechanism. Proc Natl Acad Sci USA. (2013) 110:18162–7. doi: 10.1073/pnas.1317049110 PMC3831479 24145431

[45] Bonnet N Bourgoin L Biver E Douni E Ferrari S. RANKL inhibition improves muscle strength and insulin sensitivity and restores bone mass. J Clin Invest. (2019) 129:3214–23. doi: 10.1172/JCI125915 PMC6668701 31120440

[46] Langen RC Schols AM Kelders MC Wouters EF Janssen-Heininger YM. Inflammatory cytokines inhibit myogenic differentiation through activation of nuclear factor-kappaB. FASEB J. (2001) 15:1169–80. doi: 10.1096/fj.00-0463 11344085

[47] Lee D Goldberg AL. Muscle wasting in fasting requires activation of NF-κB and inhibition of AKT/mechanistic target of rapamycin (mTOR) by the protein acetylase, GCN5. J Biol Chem. (2015) 290:30269–79. doi: 10.1074/jbc.M115.685164 PMC4683253 26515065

[48] Zhang K Zhaos J Liu X Yan B Chen D Gao Y. Activation of NF-B upregulates Snail and consequent repression of E-cadherin in cholangiocarcinoma cell invasion. Hepatogastroenterology. (2011) 58:1–7. 21510277

[49] Mii S Enomoto A Shiraki Y Taki T Murakumo Y Takahashi M. CD109: a multifunctional GPI-anchored protein with key roles in tumor progression and physiological homeostasis. Pathol Int. (2019) 69:249–59. doi: 10.1111/pin.12798 31219232

[50] Shiraki Y Mii S Enomoto A Momota H Han YP Kato T. Significance of perivascular tumour cells defined by CD109 expression in progression of glioma. J Pathol. (2017) 243:468–80. doi: 10.1002/path.4981 28888050

[51] Chuang CH Greenside PG Rogers ZN Brady JJ Yang D Ma RK. Molecular definition of a metastatic lung cancer state reveals a targetable CD109-Janus kinase-Stat axis. Nat Med. (2017) 23:291–300. doi: 10.1038/nm.4285 28191885 PMC6453542

[52] Hu WH Chang CD Liu TT Chen HH Hsiao CC Kang HY. Association of sarcopenia and expression of interleukin-23 in colorectal cancer survival. Clin Nutr. (2021) 40:5322–6. doi: 10.1016/j.clnu.2021.08.016 34536640

[53] Ohmori H Kawahara I Mori T Nukaga S Luo Y Kishi S. Evaluation of parameters for cancer-induced sarcopenia in patients autopsied after death from colorectal cancer. Pathobiology. (2019) 86:306–14. doi: 10.1159/000503037 31707381

[54] Briukhovetska D Dörr J Endres S Libby P Dinarello CA Kobold S. Interleukins in cancer: from biology to therapy. Nat Rev Cancer. (2021) 21:481–99. doi: 10.1038/s41568-021-00363-z PMC8173513 34083781

[55] De Simone V Franzè E Ronchetti G Colantoni A Fantini MC Di Fusco D. Th17-type cytokines, IL-6 and TNF-α synergistically activate STAT3 and NF-kB to promote colorectal cancer cell growth. Oncogene. (2015) 34:3493–503. doi: 10.1038/onc.2014.286 PMC4493653 25174402

[56] Assis DN. Chronic complications of cholestasis: evaluation and management. Clin Liver Dis. (2018) 22:533–44. doi: 10.1016/j.cld.2018.03.014 30259851

[57] Bolam KA van Uffelen JG Taaffe DR. The effect of physical exercise on bone density in middle-aged and older men: a systematic review. Osteoporos Int. (2013) 24:2749–62. doi: 10.1007/s00198-013-2346-1 23552825

[58] Tanishima S Morio Y. A review of minodronic acid hydrate for the treatment of osteoporosis. Clin Interv Aging. (2013) 8:185–9. doi: 10.2147/CIA PMC3578444 23440003

[59] Edwards BJ Bunta AD Lane J Odvina C Rao DS Raisch DW. Bisphosphonates and nonhealing femoral fractures: analysis of the FDA Adverse Event Reporting System (FAERS) and international safety efforts: a systematic review from the Research on Adverse Drug Events And Reports (RADAR) project. J Bone Joint Surg Am. (2013) 95:297–307. doi: 10.2106/JBJS.K.01181 23426763 PMC3748968

