# Supplementary material for: Impact of osteopenia and osteosarcopenia on the outcomes after surgery of hepatobiliary-pancreatic cancers
Source: Front Oncol. 2024 Jul 19;14:1403822. doi: 10.3389/fonc.2024.1403822 (PMC11294096; doi:10.3389/fonc.2024.1403822)
Supplement: Supplementary file 1 [file Table_1.docx]

Supplementary material 1. Detailed search strategy

((((((((Bone Density[Title/Abstract]) OR (Bone Densities[Title/Abstract])) OR (Bone Mineral Density[Title/Abstract])) OR (Bone Mineral Densities[Title/Abstract])) OR (Bone Mineral Content[Title/Abstract])) OR (Bone Mineral Contents[Title/Abstract])) OR (Osteopenia[Title/Abstract]) OR ("Bone Density"[Mesh])) OR (Osteosarcopenia[Title/Abstract])) AND ((((((((((((((((((((("Neoplasm, Pancreatic"[Title/Abstract]) OR ("Pancreatic Neoplasm"[Title/Abstract])) OR (Pancreas Neoplasms[Title/Abstract])) OR ("Neoplasm, Pancreas"[Title/Abstract])) OR ("Neoplasms, Pancreas"[Title/Abstract])) OR ("Pancreas Neoplasm"[Title/Abstract])) OR ("Neoplasms, Pancreatic"[Title/Abstract])) OR ("Cancer of Pancreas"[Title/Abstract])) OR ("Pancreas Cancers"[Title/Abstract])) OR ("Pancreas Cancer"[Title/Abstract])) OR ("Cancer, Pancreas"[Title/Abstract])) OR ("Cancers, Pancreas"[Title/Abstract])) OR ("Pancreatic Cancer"[Title/Abstract])) OR ("Cancer, Pancreatic"[Title/Abstract])) OR ("Cancers, Pancreatic"[Title/Abstract])) OR ("Pancreatic Cancers"[Title/Abstract])) OR ("Cancer of the Pancreas"[Title/Abstract])) OR ("Pancreatic Neoplasms"[Mesh])) OR ((((((((((((((((((((Carcinomas, Pancreatic Ductal[Title/Abstract]) OR (Ductal Carcinoma, Pancreatic[Title/Abstract])) OR (Ductal Carcinomas, Pancreatic[Title/Abstract])) OR (Carcinoma, Pancreatic Ductal[Title/Abstract])) OR (Pancreatic Ductal Carcinomas[Title/Abstract])) OR (Duct-Cell Carcinoma of the Pancreas[Title/Abstract])) OR (Duct Cell Carcinoma of the Pancreas[Title/Abstract])) OR (Pancreatic Ductal Carcinoma[Title/Abstract])) OR (Ductal Carcinoma of the Pancreas[Title/Abstract])) OR (Pancreatic Duct Cell Carcinoma[Title/Abstract])) OR (Carcinoma, Ductal, Pancreatic[Title/Abstract])) OR (Duct-Cell Carcinoma, Pancreas[Title/Abstract])) OR (Carcinoma, Pancreas Duct-Cell[Title/Abstract])) OR (Carcinomas, Pancreas Duct-Cell[Title/Abstract])) OR (Duct Cell Carcinoma, Pancreas[Title/Abstract])) OR (Duct-Cell Carcinomas, Pancreas[Title/Abstract])) OR (Pancreas Duct-Cell Carcinoma[Title/Abstract])) OR ("Carcinoma, Pancreatic Ductal"[Mesh])) OR (Pancreas Duct-Cell Carcinomas[Title/Abstract])) OR (((((((((((((Intraductal Neoplasms, Pancreatic[Title/Abstract]) OR (Neoplasm, Pancreatic Intraductal[Title/Abstract])) OR (Pancreatic Intraductal Neoplasm[Title/Abstract])) OR (Pancreatic Intraductal Tubulopapillary Neoplasm[Title/Abstract])) OR (Intraductal Tubulopapillary Neoplasm, Pancreatic[Title/Abstract])) OR (Pancreatic Intraductal Papillary Mucinous Neoplasm[Title/Abstract])) OR (Pancreatic IPMN[Title/Abstract])) OR (IPMN, Pancreatic[Title/Abstract])) OR (Pancreatic IPMNs[Title/Abstract])) OR (Intraductal Papillary-Mucinous Neoplasm, Pancreatic[Title/Abstract])) OR (Intraductal Papillary Mucinous Neoplasm, Pancreatic[Title/Abstract])) OR (Intraductal Papillary Mucinous Neoplasms of the Pancreas[Title/Abstract])) OR ("Pancreatic Intraductal Neoplasms"[Mesh])))) OR (((((((((((("Gallbladder Neoplasms"[Title/Abstract]) OR ("Gallbladder Neoplasm"[Title/Abstract])) OR ("Neoplasm, Gallbladder"[Title/Abstract])) OR ("Neoplasms, Gallbladder"[Title/Abstract])) OR ("Cancer of Gallbladder"[Title/Abstract])) OR ("Gallbladder Cancers"[Title/Abstract])) OR ("Gallbladder Cancer"[Title/Abstract])) OR ("Cancer, Gallbladder"[Title/Abstract])) OR ("Cancers, Gallbladder"[Title/Abstract])) ) OR ("Cancer of the Gallbladder"[Title/Abstract]) OR ("Gallbladder Neoplasms"[Mesh])) OR ((((((((((((("cholangiocellular cancer"[Title/Abstract]) OR ("Cholangiocellular Carcinoma"[Title/Abstract]) OR ("Carcinoma, Cholangiocellular"[Title/Abstract])) OR ("Carcinomas, Cholangiocellular"[Title/Abstract])) OR ("Cholangiocellular Carcinomas"[Title/Abstract])) OR ("Extrahepatic Cholangiocarcinoma"[Title/Abstract])) OR ("Cholangiocarcinoma, Extrahepatic"[Title/Abstract])) OR ("Extrahepatic Cholangiocarcinomas"[Title/Abstract])) OR ("Intrahepatic Cholangiocarcinoma"[Title/Abstract])) OR ("Cholangiocarcinoma, Intrahepatic"[Title/Abstract])) OR ("Cholangiocarcinomas, Intrahepatic"[Title/Abstract])) OR ("Intrahepatic Cholangiocarcinomas"[Title/Abstract]) OR ("Cholangiocarcinoma"[Title/Abstract]) OR ("Cholangiocarcinoma"[Mesh])) OR (((((((((("Bile Duct Neoplasm"[Title/Abstract]) OR ("Bile Duct Neoplasms"[Title/Abstract])) OR ("Neoplasms, Bile Duct"[Title/Abstract])) OR ("Bile Duct Cancer"[Title/Abstract])) OR ("Bile Duct Cancers"[Title/Abstract])) OR ("Cancer, Bile Duct"[Title/Abstract])) OR ("Cancers, Bile Duct"[Title/Abstract])) OR ("Cancer of the Bile Duct"[Title/Abstract])) OR ("Cancer of Bile Duct"[Title/Abstract])) OR ("Bile Duct Neoplasms"[Mesh]))) OR ((((((((("Biliary Tract Neoplasm"[Title/Abstract]) OR ("Neoplasms, Biliary Tract"[Title/Abstract])) OR ("Biliary Tract Cancer"[Title/Abstract])) OR ("Biliary Tract Cancers"[Title/Abstract])) OR ("Cancer, Biliary Tract"[Title/Abstract])) OR ("Cancers, Biliary Tract"[Title/Abstract])) OR ("Cancer of the Biliary Tract"[Title/Abstract])) OR ("Cancer of Biliary Tract"[Title/Abstract])) OR ("Biliary Tract Neoplasms"[Mesh]))))) OR (((((((((((((((((((((Carcinomas, Hepatocellular[Title/Abstract]) OR (Hepatocellular Carcinomas[Title/Abstract])) OR (Liver Cell Carcinoma, Adult[Title/Abstract])) OR (Liver Cancer, Adult[Title/Abstract])) OR (Adult Liver Cancer[Title/Abstract])) OR (Adult Liver Cancers[Title/Abstract])) OR (Cancer, Adult Liver[Title/Abstract])) OR (Cancers, Adult Liver[Title/Abstract])) OR (Liver Cancers, Adult[Title/Abstract])) OR (Liver Cell Carcinoma[Title/Abstract])) OR (Carcinoma, Liver Cell[Title/Abstract])) OR (Carcinomas, Liver Cell[Title/Abstract])) OR (Cell Carcinoma, Liver[Title/Abstract])) OR (Cell Carcinomas, Liver[Title/Abstract])) OR (Liver Cell Carcinomas[Title/Abstract])) OR (Hepatocellular Carcinoma[Title/Abstract])) OR (Hepatoma[Title/Abstract])) OR (Hepatomas[Title/Abstract])) OR ("Carcinoma, Hepatocellular"[Mesh])) OR ((((((((((((((((((((((Cancer, Hepatocellular[Title/Abstract]) OR (Cancer of the Liver[Title/Abstract])) OR (Liver Cancers[Title/Abstract])) OR (Cancers, Liver[Title/Abstract])) OR (Cancer, Liver[Title/Abstract])) OR (Liver Cancer[Title/Abstract])) OR (Hepatic Cancers[Title/Abstract])) OR (Cancers, Hepatic[Title/Abstract])) OR (Cancer, Hepatic[Title/Abstract])) OR (Hepatic Cancer[Title/Abstract])) OR (Hepatocellular Cancers[Title/Abstract])) OR (Cancers, Hepatocellular[Title/Abstract])) OR (Hepatocellular Cancer[Title/Abstract])) OR (Cancer of Liver[Title/Abstract])) OR (Neoplasm, Hepatic[Title/Abstract])) OR (Hepatic Neoplasm[Title/Abstract])) OR (Hepatic Neoplasms[Title/Abstract])) OR (Neoplasm, Liver[Title/Abstract])) OR (Liver Neoplasm[Title/Abstract])) OR (Neoplasms, Liver[Title/Abstract])) OR (Neoplasms, Hepatic[Title/Abstract])) OR ("Liver Neoplasms"[Mesh])) ) OR (HCC[Title/Abstract])))
