# Supplementary figures and images for: Impact of osteopenia and osteosarcopenia on the outcomes after surgery of hepatobiliary-pancreatic cancers
Source: Front Oncol. 2024 Jul 19;14:1403822. doi: 10.3389/fonc.2024.1403822 (PMC11294096; doi:10.3389/fonc.2024.1403822)

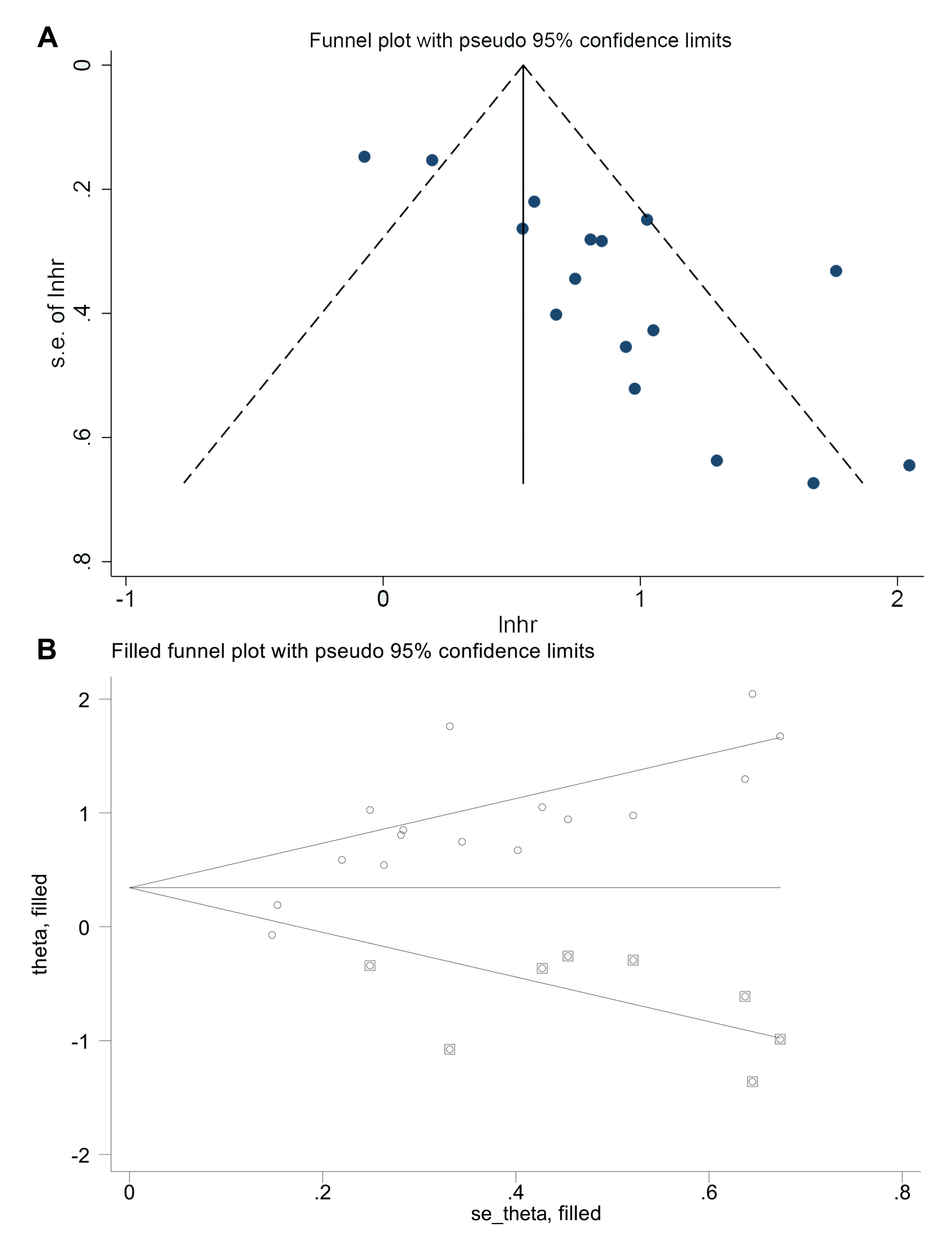

Supplement: Supplementary Figure 1 — (A) Funnel plots of the relationship between osteopenia and overall survival; These two diagonal lines are the confidence intervals for the funnel plot; the vertical line in the middle represents the combined HR values, and when unbiased (ideally), the individual studies should be equally distributed on both sides of the vertical line in an inverted funnel shape. (B) The picture of the trim-and-fill method. Theta, the effect estimate; Se_theta, the corresponding standard error; The circles represent the studies included in this meta-analysis; Boxes with circles represent additional studies of the trim and fill method. [file Image_1.tif]

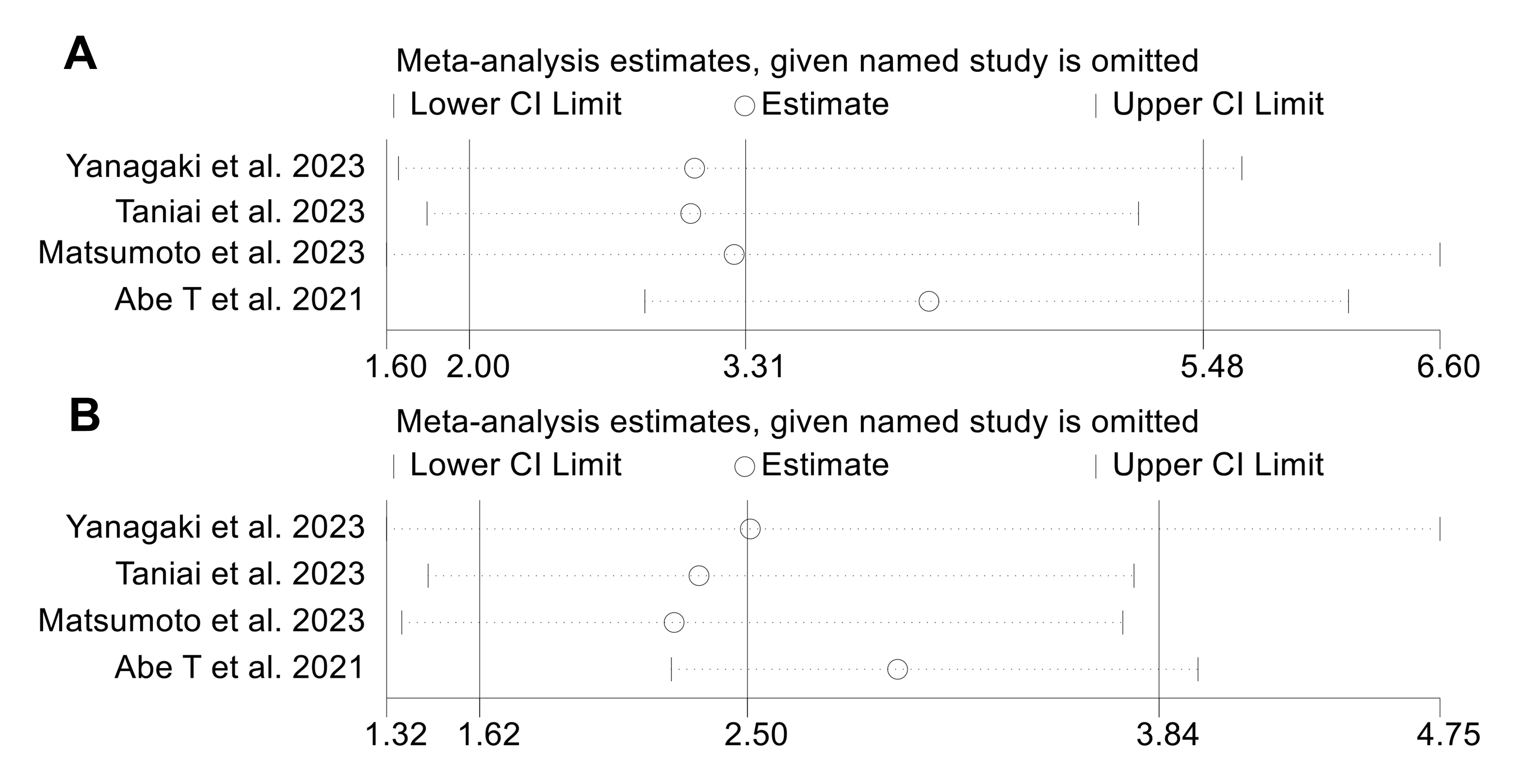

Supplement: Supplementary Figure 2 — Sensitivity analysis of the association between osteosarcopenia and overall survival (A) and recurrence-free survival (B). CI, confidence interval. [file Image_2.tif]
